# Effect of Low-Carbohydrate Diet on Beta-Hydroxybutyrate Ketogenesis Metabolic Stimulation and Regulation of NLRP3 Ubiquitination in Obese Saudi Women

**DOI:** 10.3390/nu15040820

**Published:** 2023-02-05

**Authors:** Manal Abdulaziz Binobead, Azhar Hamad Aldakhilallah, Sahar Abdulaziz Alsedairy, Laila Naif Al-Harbi, Wahidah H. Al-Qahtani, Ghedeir M. Alshammari

**Affiliations:** Department of Food Science and Nutrition, College of Food and Agricultural Sciences, King Saud University, Riyadh 11451, Saudi Arabia

**Keywords:** ketogenic diet, obesity, women, triglycerides, interleukin-1beta

## Abstract

The effects of a ketogenic diet (KD) on anthropometric indices, the lipid profile, and the benefits of the ketone body beta-hydroxybutyrate (BHB) as an inhibitor of the NOD-like receptor pyrin domain-containing 3 (NLRP3) inflammasome in obese women were investigated in this study. From January to March 2021, 23 obese adult women (*n* = 23) with an average age of 35.30 years and BMI of 33.96 kg/m^2^ followed a KD. Instructions for the KD were given to eligible participants, with a typical plan and a menu for all the main meals, snacks, and drinks permitted over seven days. They were also free to change meals according to their preferences provided that they followed the plan. The participants attended six times throughout the intervention for measurements of their anthropometric indices, BHB levels, interleukin-1beta (1L-1β) levels, and completion of a questionnaire (pre-intervention, mid-intervention, and post-intervention). Following the KD caused significant weight loss, a reduction in waist circumference and BHB levels, as well as a reduction in BMI and appetite. Cholesterol, triglycerides (TG), and high-density lipoprotein cholesterol (HDL-C) increased slightly. However, low-density lipoprotein cholesterol (LDL-C) in serum increased significantly (*p* < 0.05), and 1L-1β decreased significantly (*p* < 0.0001). The results show that the KD effectively encouraged weight loss and NLRP3 inflammasome inhibition. Based on the questionnaire results, it was found that a variety of physical symptoms, including overall energy, physical activity, mood, sleep, focus, skin conditions, and menstruation, had significantly improved.

## 1. Introduction

Despite the efforts of the medical community, obesity is a pandemic that is rapidly spreading and remains a major health problem [1]. Obesity has been linked to a variety of chronic diseases, such as type 2 diabetes, hypertension, cardiovascular disease, dyslipidemia, nonalcoholic fatty liver disease, chronic kidney disease, mood disorders, and physical disabilities [2]. Diabetes, hypertension, and obesity were all negatively impacted by the shift toward a greater reliance on processed foods, eating out more frequently, increasing the use of edible oils and sugar-sweetened beverages, declining physical activity, and an increase in sedentary behavior [3]. The startling rise in obesity rates has been linked to drastic changes in lifestyle, eating habits, socioeconomic level, and changing societal and cultural trends, as well as binge eating, fast food intake, and lack of physical activity [4].

Saudi Arabia has been Westernizing over the past few decades, and as a result, it now has one of the highest prevalence rates of obesity and overweight people [5,6]. According to a national epidemiological health survey that collected data from 17,232 Saudi household members aged 30–70, overweight and obesity are rising in Saudi Arabia, particularly among females, and they are well-known causes of coronary artery disease [7]. Additionally, Ahmed et al. [8] provided epidemiological information on the incidence of obesity in Hail, Saudi Arabia. Using data from 5000 Saudis collected from 30 primary healthcare facilities, the study revealed an overall obesity incidence of 63.6% in the Hail region. In addition, 56.2% of men and 71% of women in the area were obese.

Obesity is associated with the emergence of a chronic low-grade inflammatory response brought on by adipocyte enlargement and fat mass expansion, which allows macrophages to infiltrate adipose tissue [9]. As a result, proinflammatory cytokines, such as interleukin-1beta (IL-1β) and interleukin-1sigma (IL-1σ), are generated [10], and these have been linked to the onset of obesity-related insulin resistance [11]. Although diet composition has a direct impact on ad libitum energy intake, the rise in obesity around the world has prompted an urgent search for effective weight-loss methods [12]. The KD has recently been favored as a weight loss method because of its effectiveness in lowering TG and insulin levels and improving physical performance while having no negative impact on total cholesterol or LDL levels [13].

Regulated calorie intake combined with the ketogenic diet (KD) has gained popularity in recent years after being recommended as a successful weight loss strategy [14]. The KD has been demonstrated to be an effective treatment for reducing some cardiovascular risk factors, enhancing glycemic control, and combating obesity in short to medium term [15]. A 12-week KD resulted in significant weight loss and improved physical performance, cognitive function, eating, and metabolization. On the other hand, significant reductions in insulin and glucose levels together with decreased appetite were detected in a study performed by an interdisciplinary team involving 38 obese adults (13 men, 25 women) aged 37 ± 7 years with a BMI of 36.1 ± 5.6 kg [16]. Despite the diet’s beneficial effect on HDL-C, concurrent increases in LDL-C and very low-density lipoproteins (VLDL) may increase cardiovascular risks [17]. Patients with diabetes who are on insulin or oral hypoglycemic agents, on the other hand, may experience severe hypoglycemia if their medication regimen is not properly managed during the initiation of KD [17]. Additionally, patients with liver failure, pancreatitis, inherited disorders of fat metabolism, or deficiency in primary carnitine, carnitine palmitoyl transferase, carnitine translocase, porphyria, or pyruvate kinase should not consume a diet that is restricted or contraindicated [18]. The phrase “keto flu” has been used to describe typical short-term adverse effects of starting KD and includes symptoms such as decreased exercise tolerance, fatigue, headache, dizziness, nausea, and vomiting [18].

The inflammatory NOD-like receptor protein 3 (NLRP3) complex is a tightly controlled inflammatory response to both internal and external cellular abnormalities and microbial components [19]. It is a powerful innate immune sensor that is activated by several structural molecular patterns associated with damage (DAMPs), including toxins, adenosine triphosphate (ATP), excessive glucose, ceramides, amyloids, urates, and cholesterol crystals [19]. Cysteinyl protein aspartate-specific-1 (caspase-1) maturation, fast cleavage, and IL-1β and IL-1σ secretion occur when inflammatory NLRP3 is activated by elements such as ATP and monosodium urate crystals (MSU) [20]. IL-1β and IL-1σ are members of the interleukin-1 (IL-1) family, which are important proinflammatory regulators in the brain and operate as the main signal to induce the activation of inflammatory NLRP3 [21]. IL-1β is involved in triggering systemic and local responses to infection and immunological challenges by creating an “acute phase response” marked by fever, acute-phase protein production, and leukopenia [22]. Despite lacking the pyrogenic action of IL-1β, IL-1σ is implicated in generating numerous secondary inflammatory cytokines [23]. The KD was predicted to have a positive effect on the parameters studied. Therefore, in this study, we set out to determine the effect of a very low-carbohydrate diet or a KD on the anthropometric indices and lipid profiles of obese Saudi women, and the effect of KD-induced beta-hydroxybutyrate (BHB) stimulation on the inhibition of the NLRP3 inflammasome and IL-1β.

## 2. Materials and Methods

### 2.1. Participants

Advertisements on “WhatsApp” were used to recruit participants from the Saudi Arabian city of Buraydah. The eligibility requirements for the participants were as follows: Saudi women in their 20 s to 50 s who were “physically well”, had a BMI greater than 30 kg/m^2^, were free of disabilities and chronic diseases, and were prepared to consume the meals included in the study. At the Nutrition Clinic of the Buraydah Central Hospital, all participants underwent medical evaluations to exclude those with cardiovascular disorders, uncontrolled hypertension, impaired kidney function, liver failure, or who were pregnant or breastfeeding. All the participants signed consent forms.

### 2.2. Study Design

From January to March 2021, the study was carried out at the Buraydah Central Hospital. This was a repeated-measurement uncontrolled intervention research. The prospective participants listened to a presentation on a very low carbohydrate or ketogenic diet, which provided details of the diet, the significance of nutritional composition, recommended eating times each day, the benefits of the diet, and the high-fat diet. All potential participants were asked to consume a KD for eight weeks while maintaining their normal physical activity levels. The study protocol and potential risks were fully explained to the participants before their written consent was obtained. Participants were asked to attend six sessions during the intervention period, at baseline (pre-intervention), weeks 1, 2, and 4 (mid-intervention), week 6, and week 8 (post-intervention) for an assessment of their body weight, height, BMI, waist circumference, and BHB levels.

The session was held at the Nutrition Clinic of the Buraydah Central Hospital. A very low carbohydrate or ketogenic diet with caloric restriction was used as the experimental intervention. At the start of the intervention, all participants attended a two-hour lecture about a KD, nutritional composition, the proper times to eat, and the beneficial effects of a KD, as well as an educational session about their prescribed diet plan. The participants were given a list of very low-carbohydrate foods to choose from in order to consume 20 g of carbohydrates per day. They were provided with a typical plan and a menu for all main dishes, snacks, and drinks allowed for seven days, with instructions to limit their carbohydrate intake to 5–10% of energy intake (EI) and to derive at least 75% of it from fat, incorporate a protein intake of 20%, and follow a very low-energy diet with a deliberate caloric deficit (1200–1500 kcal). Unlimited quantities of meat, poultry, fish, shellfish, and eggs were permitted, as were two cups of salad and vegetables per day, one cup of low-carbohydrate vegetables per day, four ounces of hard cheese, and limited amounts of cream, avocado, olives, and lemon juice. Transformed fats were prohibited, but fats and oils were not. Detailed explanations of how to apply the KD principles in practice and appropriate tracking and monitoring procedures were provided. The participants were instructed not to use drugs or antioxidants to eliminate the possibility of confounding effects. BHB levels were measured weekly to assess adherence to the dietary regimen.

### 2.3. Questionnaires

A structured questionnaire validated by nutrition experts was designed for use before and after participation. Each questionnaire consisted of three parts:

#### 2.3.1. Pre-Participation Questionnaire

The first section contained demographic data (name, age, educational status, and income) and anthropometric measurements (weight, BMI, and waist circumference). The second section covered diseases or allergies caused by a specific food. The third section covered their diet, appetite, physical activity, and nutritional knowledge, particularly of the KD.

#### 2.3.2. Post-Participation Questionnaire

The first section of the questionnaire used after the diet included anthropometric measurements (weight, BMI, and waist circumference). The second part focused on the participant’s level of commitment to the program and their diet in terms of health, appetite, and the number of meals they consumed per day. The third section explored the psychological state of participants after completing the KD, including mood, focus, general energy, skin condition, menstruation, and athletic activity.

### 2.4. Measurements of Subjective Appetite

In accordance with Flint et al. [24], with slight modification, the participants’ subjective appetite was assessed using a standard appetite questionnaire, which included the following questions for their general appetite, such as “please rate the appetite after sharing as low, normal, or high while that for sweets to be non-existent, low, average, high or very high”. The results are presented as frequencies and percentages.

### 2.5. Anthropometric Measurement

Anthropometric measurements were taken between 8 a.m. and 11 a.m., after an overnight fast (8 to 10 h), in standardized conditions and by the same examiner at the beginning and end of the intervention. The heights, BMIs, and waist circumferences of the participants were measured while they were standing with their heads and knees straight, wearing light clothing and no shoes or headgear. Weight (kg)/height^2^ (m^2^) was the formula used to calculate BMI (kg/m^2^). Obesity was defined as having a BMI greater than 30 [16].

### 2.6. Lipid and Lipoprotein Profiles

Blood analysis was performed twice: once before (0 weeks) and once after the end of the study (8 weeks). After an overnight fast of at least 8 h, blood samples of 5 mL were collected and coagulated for at least 30 min at room temperature before being centrifuged at 3500 rpm for 7 min. For each sample, 1 mL of separated serum was placed in a microtube and frozen. The frozen serum was analyzed by the Buraydah Central Hospital Laboratories using a Chemistry Analyzer (Beckman Coulter UniCel DxC 600; Beckman Coulter, Brea, CA, USA). In accordance with Choi et al. [25], the analysis included the total cholesterol, triglyceride, LDL-C, and HDL-C levels.

### 2.7. Identification of Levels of Ketone Bodies

Ketosis was identified using a portable meter (FreeStyle Optium Neo Blood Glucose and Ketone Monitoring meters) to measure the ketone bodies, specifically beta-hydroxybutyrate (BHB), in the capillaries. All capillary ketonemia diagnoses were performed after an overnight fast of 8 to 10 h, as with the anthropometric assessments. Each participant performed these measurements in five sessions (at 1, 2, 4, 6, and 8 weeks) throughout the KD diet, and the corresponding values were reviewed on the machine memory to control adherence [26].

### 2.8. Quantification of Cytokine IL-1β

Cloud-Clone Corporation (CCC, Katy, TX, USA) provided the enzyme-linked immunosorbent assay (ELISA) kits, including an IL-1β kit. The ELISA was carried out following the manufacturer’s instructions. Immediately after the blood collection, the serum was separated by centrifugation for 20 min in 5 mL collection tubes, and the samples were stored in aliquots at −20 °C for later use. Before use, the samples were brought to room temperature (18–25 °C), and 100 μL of serum was added to the appropriate wells and incubated for one hour at 37 °C. After removing the excess cytokines, 100 μL of the antibody solution (A) was added to the wells and incubated for one hour at 37 °C. Following incubation, the wells were washed three times with a washing solution, then 100 μL of the antibody solution (B) was added to the wells and incubated for one hour at 37 °C. After repeated washing, 90 μL of the substrate solution was added to each well and incubated for 30 min. The reaction was stopped after incubation by adding a stop solution to each well. Finally, cytokine levels were estimated using a microplate reader to measure absorbance at 450 nm [27].

### 2.9. Statistical Analysis

For data entry and analysis, IBM’s Statistical Package for Social Science (SPSS) version 23 (Chicago, IL, USA) was used. For the quantitative data analysis, basic frequencies and percentages and the means and standard deviations were used to describe and compare responses among individuals. Paired *t*-tests and chi-square tests were used to compare the means at baseline and in the weeks before and after. Repeated analysis of variance measurements was used to investigate the effects of the KD on changes in the anthropometric measurements over time. *p* < 0.05 was chosen as the statistical significance level [25].

## 3. Results

### 3.1. Demographic Characteristics and Changes in Anthropometric Indices

Out of 40 volunteers, 23 obese females aged from 18 to 48 years were found to be eligible for the study and passed the preliminary tests. Table 1 shows the demographic characteristics of the participants. The majority of participants (65.2%) were married, 73.9% were university graduates, and 82.6% had an average income. The participants were in good health and had no food allergies that interfered with their diet. Table 2 shows the changes in the anthropometric indices of participants (*n* = 23) before and after following the ketogenic diet. The average age and height of the participants were 35.3 years and 156.83 cm, respectively. The majority of the participants were overweight, with an average body mass index (BMI) of 33.96 and a waist circumference of 99.04 cm. The nutritional intervention resulted in a significant (*p* < 0.0001) reduction in body weight, BMI, and waist circumference. After the first month, the body weight of all participants decreased progressively throughout the study. BMI and waist circumference decreased significantly (*p* < 0.0001) and continued to decrease progressively throughout the study.

### 3.2. Changes in Blood Ketone Body β-Hydroxybutyrate (BHB), Blood Lipids, and IL-1β

Depending on the baseline, the level of ketone bodies in the blood increased significantly (*p* < 0.0001) at the first session (1.235 mmol/L) and continued to increase up to week 8 (0.2565 mmol/L) as shown in Table 3. In terms of BHB, there were minor differences among the sessions. As shown in Table 4, the ketogenic diet increased the total cesterol slightly but not significantly. Triglyceride levels fell slightly but not significantly from 1.04 to 0.91, as did HDL-C levels, which fell from 1.35 to 1.33. However, there was a significant (*p* < 0.05) increase in LDL-C. The ketogenic diet significantly (*p* < 0.0001) decreased the IL-1β cytokine from 121.38 to 20.70 ng/mL.

### 3.3. Changes in Lifestyle and Body Symptoms

Table 5 shows the frequency distribution of participants’ commitment to the program and diet and the number of meals eaten per day and the psychological state before and after completing the ketogenic diet (KD). A structured questionnaire assessed the lifestyle changes and physical symptoms of the participants. The participants noticed a significant difference in their diets before and after following the KD, and the comparison revealed that 56.52% of the participants said their diet was almost healthy before starting the KD, but 86.96% said their diet was healthy after starting the KD. Before starting the KD, 52.17% of the participants rated two meals as the main meals, but after starting the KD, this percentage increased to 56.52% of the participants, who also had three main meals. According to the questionnaire, the skin conditions of the participants improved significantly, as evidenced by a high percentage (60.87%) who stated that the improvement in their skin was excellent after starting the KD, compared to 4.35% who stated this before starting the KD. However, 60.87% of the participants said their skin condition was good before following the KD, whereas 39.13% said it was good after following the KD. None of the participants believed their skin was in poor condition after following the KD, but 34.78% reported poor skin before starting the KD. About 65.22% of the participants reported a significant improvement in menstruation after starting the KD, and 56.52% reported that it was good even before starting the KD. Approximately 39.13% of the participants reported that their menstruation was bad but improved after following the KD. A significantly higher percentage of the participants (69.56%) reported that their general energy was excellent after following the KD, compared to 52.17% who reported that it was low before beginning the KD. Before starting the KD, approximately 39.13 and 60.87% reported that their levels of physical activity were average and low, respectively, but after starting the KD, 39.13 and 60.87% reported that their levels of physical activity were high and average, respectively. Psychologically, after following the KD, the mood of the participants improved significantly, with 78.26% reporting that their mood was excellent. Sleep conditions also improved significantly and were reported to be excellent (73.91%). Furthermore, 56.52% of the participants agreed that the KD had improved their ability to focus. When comparing appetite before and after the KD, there was a decrease in the average general appetite and appetite for sweets (56.52% and 60.87%, respectively).

## 4. Discussion

This study aimed to find out how a very low-carbohydrate diet (KD) affected the anthropometric indices of obese Saudi women and how beta-hydroxybutyrate (BHB) stimulation affected the inhibition of the NLRP3 inflammasome and IL-1β. The majority of participants were married, had a college education, and earned an average income. Even though the majority of participants were young, educated, healthy, and had a decent monthly income, their knowledge of food regimes was very poor. A person with a good education can control their eating habits to promote moderate rather than excessive consumption and to highlight healthy food options rather than rigid diet regimens. This could have been because most participants lacked sufficient free time to attend symposiums or lectures concerning diet.

Our results demonstrate that the KD intervention led to a significant reduction in the participants’ weights, BMIs, and waist circumferences, as well as a drop in IL-1β levels, as reported by Zhu et al. [27]. The investigation, therefore, supports our main hypothesis. The results are consistent with the research on obese individuals, demonstrating how low-carbohydrate and ketogenic diet therapy efficiently reduces body fat while dramatically boosting skeletal muscle mass [28,29]. In our study, every participant lost weight, reduced their BMI, and had a smaller waist circumference. One study also found that there was a correlation between weight loss and waist circumference with an increase in ketone bodies in the body during a ketogenic diet. This correlation is due to the mechanism of ketogenic bodies, which results from the decomposition of stored fats to be produced as energy for the body [30]. Additionally, a study of obese patients revealed that adherence to a ketogenic diet was associated with reductions in weight, BMI, and waist circumference [16,25].

Biochemically, the results reveal that although the total cholesterol, TG, and HDL levels all slightly improved, high levels of LDL-C were found following the intervention. This was probably because blood LDL-C levels are sensitive to higher fat intake, but this was likely to reduce in subsequent weeks as the diet in this study only lasted two months, as is customary. An experimental ketogenic study supported our findings that a low-carbohydrate, high-fat diet (LCKD) with supplementation improved blood lipid subgroups but had no effect on the total LDL-C other than to change it from small dense LDL-C to large buoyant LDL-C, which may reduce the risk of cardiovascular disease [31].

According to the study by Noakes and Windt [32], a high-fat, low-carbohydrate diet has distinct cholesterol characteristics, such as steady increases in LDL-C and HDL-C cholesterol, compared to a low-fat, higher-carbohydrate diet [33]. On the other hand, one study found a significant rise in high-density lipoprotein cholesterol and a link between ketosis and reduced levels of total cholesterol, triglycerides, and low-density lipoprotein cholesterol [34]. Because of the lack of insulin, the ketogenic diet has been shown to have the opposite impact by lowering cholesterol production and carbohydrate consumption [35]. Compared to low-fat diets, high-fat meals have been shown to significantly positively impact TG, lowering total cholesterol and LDL-C while increasing HDL-C [36]. The discrepancy between our study and other studies can be attributed to the fact that the majority of other studies were long-term, whereas the current study only lasted for two months, which is a typical duration.

The results of this study demonstrate that dietary changes through the use of a KD considerably raised the levels of BHB in the serum of participants, which produced several favorable effects, including weight loss, a smaller waist circumference, and the alleviation of several symptoms. Notably, the intervention decreased the level of IL-1β, a cytokine that triggers the immune system to combat NLRP3, which when its level is increased, results in a range of inflammatory illnesses. According to a prior study that concurred with this one, elevated BHB levels prevented the neutrophils of elderly subjects from secreting IL-1β, did not worsen their condition, and instead decreased their bacterial burden [37]. According to the study by Goldberg et al. [37], BHB has a strong potential to prevent inflammatory assaults such as gout. Several inflammatory and autoimmune illnesses, such as colitis and experimental allergic encephalitis, have been demonstrated to benefit from treatment with BHB, which is also a crucial mediator of amino acid and fatty acid catabolism [38,39].

The findings show that the NLRP3 inflammasome in macrophages is independently suppressed by fasting, exercise, and the ketogenic diet-induced metabolite BHB. While preserving ATP for ketone-dependent organs, including the brain and heart, metabolic signals such as BHB may decrease innate immune responses in ketogenic diet settings. The findings also illuminate the immunological functions of metabolic signals such as BHB, suggesting that dietary or pharmaceutical therapies to increase BHB without inducing a general starvation response may lessen the severity of NLRP3-mediated chronic inflammatory disorders and have a variety of beneficial effects.

Genetic, environmental, metabolic, and behavioral variables all contribute to obesity. It has been thought that food addiction is likely to be a contributory factor to obesity and weight gain following weight-loss therapy [40]. Therefore, maintaining control over these factors is essential for the effectiveness of weight-loss therapy. In reality, patients will begin to suffer a variety of unpleasant symptoms within a few days of beginning a reduced-calorie diet, including hunger, an unbalanced appetite, a craving for sweets, sadness, bad humor, and in some cases, mild depression. The results from the questionnaires completed by participants revealed no adverse effects from following the ketogenic diet, as their overall hunger and sweet cravings significantly decreased, and they reported feeling more satisfied.

This finding is consistent with a study [41] that examined the complex relationship between the ketogenic diet and food intake control. We observed that participants in our study improved their health over time and distributed their meals evenly throughout the day, which was correlated with a reduction in hunger and cravings for sweets. The idea that a KD improves general anxiety ratings and enhances mood and focus is only partially supported by limited research [42,43]. Another issue for obese people is physical activity. However, in this study, as the total energy levels of participants improved, so did their physical activity. According to Fletcher et al. [44], increasing aerobic fitness also increases the maximum fat oxidation capacity, which is further independently boosted by an increased dietary intake.

The sleep conditions of the participants improved, which is significant because it is connected to morbidity and death linked to obesity and is crucial for health [45]. The participants in the study also experienced positive psychological changes, such as better skin and menstrual health, which inspired some participants to persist with the program. The vital signs of the participants were measured, and the results revealed a connection between elevated ketone levels, a healthy appetite, and weight loss, as well as an improvement in the participants’ vital indicators. Menstruation in women and a high index of quality sleep were also positive psychological traits [26]. Furthermore, a shift from unhealthy to healthy eating habits was observed. Following the intervention, all participants became more consistent in their meal distribution, with changes in the number of meals leading to fewer meals because of increased BHB and decreased appetite. Rapid improvements in skin conditions and menstruation accompanied this. Despite instructions to maintain their normal activity, the average physical activity of all the participants increased in tandem with their overall energy levels. There was an improvement in mood, sleep, and focus.

## 5. Study Limitations

This study has some limitations. First, because this was a two-month short-term intervention, its long-term impact, and safety could not be determined. Second, although the ketogenic diet used in this trial had some positive effects on weight loss and inflammation reduction, compliance was low, and the drop-out rate was high, necessitating strict monitoring. Third, the study sample size was small because it focused on a specific region of Saudi Arabia, making it difficult to cover the entire country, and there was no control group. Fourth, in order to evaluate the effects of the KD, the study did not include a control group that consumed an isocaloric but non-ketogenic diet. For comparison, data from the same participants prior to starting the diet were used. Another problem is that the results of this study cannot be compared with a standard low-calorie nonketogenic diet.

## 6. Conclusions

In conclusion, a KD was conducted for 8 weeks to track participants’ progress in terms of their health. The study discovered that a KD induced by very low carbohydrate intake significantly reduced the weights, BMIs, and waist circumferences of the participants. Furthermore, there was a significant increase in the level of BHB in the participants’ serum, and the level of IL-1β was significantly decreased, demonstrating the inhibition of NLRP3-mediated chronic inflammation diseases. After the intervention, the total cholesterol, TG, and HDL-C levels improved slightly, but the LDL-C levels increased slightly. The KD positively impacted appetite suppression, fullness maintenance, and improvements in health status, physical activity, skin conditions, menstruation, sleeping, mood, and focus. The findings shed light on the immunological functions of metabolic signals such as BHB, implying that dietary or pharmaceutical therapies to increase BHB without inducing a general starvation response may alleviate the severity of NLRP3-mediated chronic inflammatory disorders, resulting in a variety of beneficial symptoms. Our findings support short-term KD diets as appropriate interventions to combat obesity in adults; however, due to the complexity of the potential mechanisms, their interactions, and the lack of data from controlled long-term studies (>1 year), an evidence-based recommendation to support KD diets as a preventive measure to help reduce cardiovascular risks or type 2 diabetes risks is premature. Furthermore, because transient unfavorable effects involving raised LDL-C have been reported, recommendations to consume the healthiest fat sources and reduce saturated fats should be made with caution. Diabetes patients who follow this diet should be under close medical supervision or capable of adjusting their medication.

## Figures and Tables

**Table 1:** Frequency distribution of participants (*n* = 23) according to demographic characteristics.

Variable	Frequency	Percent	*p*-Value *
Marital status			
Single	6	26.1	<0.001
Married	15	65.2	
Divorced	2	8.7	
Education			
High school	1	4.3	<0.01
University level	17	73.9	
Diploma	3	13	
Postgraduate studies	2	8.7	
Financial status			
High income	4	17.4	<0.001
Average income	19	82.6	
Medical condition			
Diabetes	1	4.3	<0.042
Irregular blood pressure	1	4.3	
Healthy	21	91.3	
Food allergy			
Yes	2	8.7	<0.001
No	21	91.3	

* chi-square test.

**Table 2:** Changes in anthropometric indices of participants (*n* = 23) before and after intake of the ketogenic diet.

Anthropometric Indices	Before Ketogenic Diet	After Ketogenic Diet	*p*-Value
Weight (kg)	84.7 (±12.26)	77.00 (±11.36)	<0.0001
Waist circumference (cm)	99.04 (±11.62)	84.83 (±7.59)	<0.0001
BMI	33.96 (±4.436)	30.91 (±4.209)	<0.0001

Data were presented as mean ± SD. Statistical significance was set at (*p* < 0.0001). Note: BMI, body mass index; SD, standard deviation.

**Table 3:** Levels of ketone body β-hydroxybutyrate (BHB, mmol/L) of participants (*n* = 23) before and after intake of the ketogenic diet.

Test Details	Mean 1	Mean 2	Mean Difference	*p*-Value
Baseline vs. 1 week	0.2565 (±0.09)	1.235 (±0.19)	0.979 ****	<0.0001
Baseline vs. 2 weeks	0.2565 (±0.09)	1.357 (±0.29)	1.101 ****	<0.0001
Baseline vs. 4 weeks	0.2565 (±0.09)	1.504 (±0.09)	1.248 ****	<0.0001
Baseline vs. 6 weeks	0.2565 (±0.09)	1.339 (±0.11)	1.083 ****	<0.0001
Baseline vs. 8 weeks	0.2565 (±0.09)	1.257 (±0.21)	1.001 ****	<0.0001

Data were presented as mean ± SD, and paired *t*-test was used to compare the changes. Statistical significance was set at **** (*p* < 0.0001). Note: BHB, β-hydroxybutyrate; SD, standard deviation.

**Table 4:** Changes in blood lipids (mmol/L) and serum levels of IL-1β (ng/mL) of participants (*n* = 23) before and after intake of the ketogenic diet.

Parameter	Before Ketogenic Diet	After Ketogenic Diet	Mean Difference	*p*-Value
Cholesterol	4.76 (±0.87)	4.99 (±1.07)	−0.22409	0.151
Triglycerides	1.04 (±0.71)	0.91 (±0.63)	0.13091	0.198
HDL-C	1.35 (±0.23)	1.33 (±0.27)	0.01818	0.710
LDL-C	2.93 (±0.71)	3.26 (±0.94)	−0.33182 *	0.024
IL-1β	121.38 (±9.076)	20.7 (±4.83)	100.68 **	<0.0001

Data were presented as mean ± SD, and paired *t*-test was used to compare the changes. For lipid profile, statistical significance was set at * (*p* < 0.05). For IL-1β, statistical significance was set at ** (*p* < 0.0001).

**Table 5:** Frequency distribution of the commitment of participants to the program and diet and the number of meals eaten per day and psychological state after completing the ketogenic diet (KD).

Item	Character	Before KD			After KD		
		Frequency	Percentage	*p*-Value	Frequency	Percentage	*p*-Value
Ketogenic diet	Healthy	1	4.35	<0.001	20	86.964	<0.001
	Almost healthy	13	56.52		3	13.04	
	Completely unhealthy	9	39.13		0	0	
Rate of meals	Three main meals	8	34.78	<0.036	10	43.48	<0.041
	Two main meals	12	52.17		13	56.52	
	One main meals	3	13.04		3	13.04	
Skin condition	Bad	8	34.78	<0.047	0	0	<0.001
	Good	14	60.87		9	39.13	
	Excellent	1	4.35		14	60.87	
Menstruation	Bad	9	39.13	<0.05	0	0	<0.001
	Good	13	56.52		8	34.78	
	Excellent	1	4.35		15	65.22	
General energy	Low	12	52.17	<0.211	0	0	<0.001
	Good	10	43.48		7	30.43	
	Excellent	1	4.35		16	69.56	
Physical activity	High	0	0	<0.049	9	39.13	<0.001
	Average	9	39.13		14	60.87	
	Low	14	60.87		0	0	
Mood	Bad	6	26.09	<0.001	0	0	<0.021
	Average	17	73.91		5	21.74	
	Excellent	0	0		18	78.26	
Sleep	Excellent (deep)	0	0	<0.451	17	73.91	<0.001
	Well	11	47.83		6	26.09	
	Bad	12	52.17		0	0	
Focus	High	1	4.35	<0.031	13	56.52	<0.001
	Average	17	73.91		10	43.48	
	Low	5	21.74		0	0	
Average general appetite	Normal	6	26.09	<0.021	9	39.13	<0.001
	High	17	73.91		1	4.35	
	Low	0	0		13	56.52	
Average appetite for sweets	Non-existent	0	0	<0.011	4	17.39	<0.001
	Low	0	0		14	60.87	
	Average	12	52.17		5	21.74	
	High	4	17.39		0	0	
	Very high	7	30.43		0	0	

## Data Availability

The datasets used and analyzed during the current study are available from the corresponding author upon reasonable request.
